# Predicting the Mechanical Properties of RCA-Based Concrete Using Supervised Machine Learning Algorithms

**DOI:** 10.3390/ma15020647

**Published:** 2022-01-15

**Authors:** Meijun Shang, Hejun Li, Ayaz Ahmad, Waqas Ahmad, Krzysztof Adam Ostrowski, Fahid Aslam, Panuwat Joyklad, Tomasz M. Majka

**Affiliations:** 1 School of Architetrue and Civil Engineering, Changchun Sci-Tech Unversity, Changchun 130600, China; 2 Jilin Northeast Architectural and Municipal Engineering Design Institute Co., Ltd., Changchun 130062, China; lihejun0720@sina.com; 3 Department of Civil Engineering, COMSATS University Islamabad, Abbottabad 22060, Pakistan; waqasahmad@cuiatd.edu.pk; 4 Faculty of Civil Engineering, Cracow University of Technology, 24 Warszawska Str., 31-155 Cracow, Poland; krzysztof.ostrowski.1@pk.edu.pl; 5 Department of Civil Engineering, College of Engineering in Al-Kharj, Prince Sattam Bin Abdulaziz University, Al-Kharj 11942, Saudi Arabia; f.aslam@psau.edu.sa; 6 Department of Civil and Environmental Engineering, Faculty of Engineering, Srinakharinwirot University, Nakhonnayok 26120, Thailand; panuwatj@g.swu.ac.th; 7 Department of Chemistry and Technology of Polymers, Faculty of Chemical Engineering and Technology, Cracow University of Technology, Warszawska 24, 31-155 Cracow, Poland; tomasz.majka@pk.edu.pl

**Keywords:** mechanical properties, aggregate, concrete, compressive strength, split tensile strength, fiber

## Abstract

Environment-friendly concrete is gaining popularity these days because it consumes less energy and causes less damage to the environment. Rapid increases in the population and demand for construction throughout the world lead to a significant deterioration or reduction in natural resources. Meanwhile, construction waste continues to grow at a high rate as older buildings are destroyed and demolished. As a result, the use of recycled materials may contribute to improving the quality of life and preventing environmental damage. Additionally, the application of recycled coarse aggregate (RCA) in concrete is essential for minimizing environmental issues. The compressive strength (CS) and splitting tensile strength (STS) of concrete containing RCA are predicted in this article using decision tree (DT) and AdaBoost machine learning (ML) techniques. A total of 344 data points with nine input variables (water, cement, fine aggregate, natural coarse aggregate, RCA, superplasticizers, water absorption of RCA and maximum size of RCA, density of RCA) were used to run the models. The data was validated using k-fold cross-validation and the coefficient correlation coefficient (R^2^), mean square error (MSE), mean absolute error (MAE), and root mean square error values (RMSE). However, the model’s performance was assessed using statistical checks. Additionally, sensitivity analysis was used to determine the impact of each variable on the forecasting of mechanical properties.

## 1. Introduction

Recently, the use of RA in concrete is gaining favour in the field of research, which gives not only environmentally friendly concrete but also shows satisfactory performance towards the mechanical properties of concrete [1,2]. In the previous decades, the production and utilization trend of sustainable concrete has been significantly increasing due to the high demand of the construction industries [3,4]. The production of concrete is now approximately 1t per human in a one-year period [5]. However, the considerable amount of concrete production fulfills the requirement of construction industries and negatively impacts the environmental conditions [6,7,8,9]. The concrete and aggregates production leads to the emission of carbon dioxide, CO_2_ gas, dust, and other harmful gases, which ultimately results in environmental pollution [10,11,12]. The demand for waste concrete is also increasing because of natural disasters such as earthquakes around the world, leading to serious environmental problems [13,14,15,16]. RCA concrete is considered as one of the potential solutions to reduce the utilization rate of the resources produced naturally and uses the waste concrete appearing from natural disasters, also from the demolition of construction [17,18]. Although the utilization of RCA in concrete is limited due to low strength, low modulus of elasticity, and high deformation, the desired strength can be achieved by adopting the suitable mix design [19].

The applications of the RCA in concrete can significantly enhance the various properties of concrete by adopting smart techniques of adding other suitable materials to it. Recently, the modern approaches of ML for anticipating results in the field of civil engineering are gaining popularity worldwide. Normally, when it comes to forecasting concrete strength, it normally requires 28 days to achieve its desired strength. The different types of ML approaches may applied to forecast the different properties of concrete without consuming time and money. There are multiple types of ML approaches that are normally applied to forecast the required output such as DT, ANN, and GEP. De-Cheng et al. [20] applied an adaptive boosting approach for the anticipation of CS of concrete in which 1030 data bases were utilized to run the required model and reported 98% accuracy compared with the actual result. Dong et al. [21] used the ANN model for high-performance concrete, and they also used Monte Carlo simulation to forecast the behavior of high-strength concrete. Muhammad et al. [22] employed GEP to foretell the concrete’s strength containing bagasse ash; the predicted accuracy was reported to be more than 80%, indicating better performance. Aliakbar et al. [23] indicated the new formulation for the mechanical properties of RA-based concrete with the help of GEP, and they also analyzed that the prediction level was close to the actual results. They investigated the CS, flexural strength, and STS from the retrieved data. Taihao et al. [24] represented their work on the application of ensemble ML techniques for the forecast and optimization of young’s modulus, having RA concrete, the RF, and SVM employed on data for prediction, which shows the accurate prediction of the outcome.

The focus of this research is based on the prediction of two properties (STS and CS) of concrete containing RCA via supervised ML algorithms [25]. The performance of both models was analyzed and compared to evaluate the better performer for the prediction of results. The accuracy level between the real and anticipated output was observed from the coefficient correlation (R^2^) value, and a higher value gives the impressive performance of the employed model. The AdaBoost technique was employed for optimization via producing 20 sub-models to obtain a higher R^2^ value [26]. The application of these ML algorithms is to compare the predictive evaluation of each approach. The significance of this study is to determine the effect of the input factors used to anticipate the mechanical characteristics of concrete and the predictive accuracy of both methodologies. The research is innovative in that it uses the type of ML techniques and individual (DT) and ensemble (AdaBoost) ML algorithms to forecast the two outcomes (CS, STS) of recycled coarse aggregate concrete (RCA). The statistical application of checks was applied to analyze the nature of both techniques. In addition, the sensitivity analysis was also incorporated, which indicates the performance level of each input parameter for the anticipation of both STS and CS.

## 2. Methodology and Description of Data

**Figure 1:**
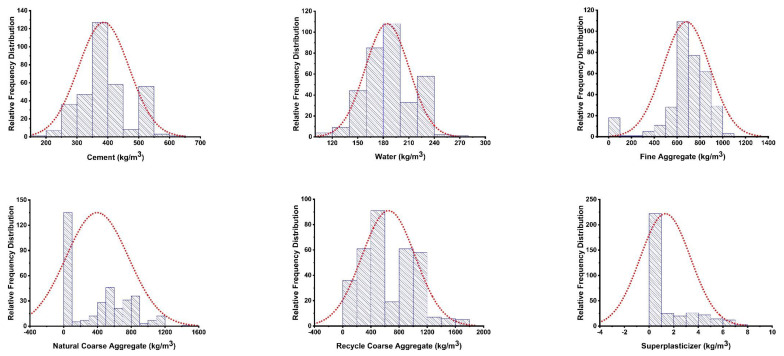

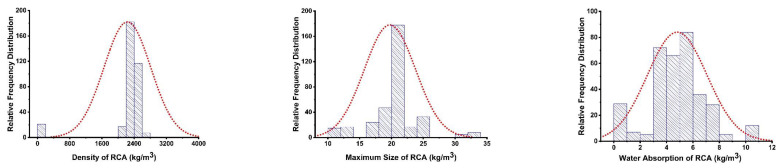
Histograms of the input parameters showing the relative frequency scattering.

**Figure 2:**
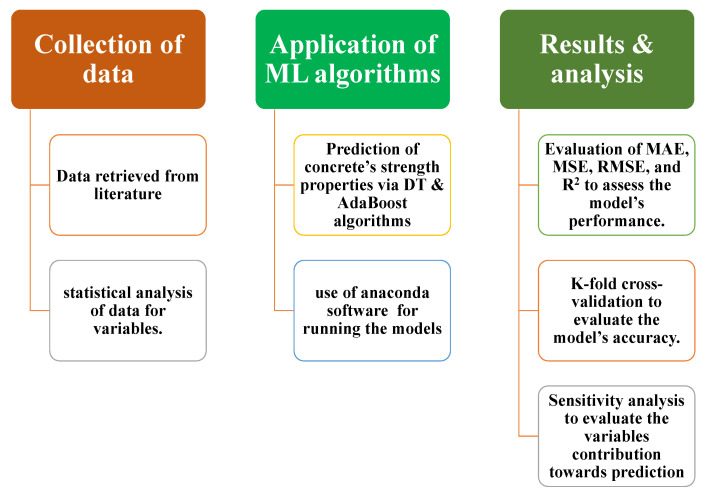
Flow chart of the research program.

The model’s performance is based on the input variables and the number of databases used to run the model. The parameters used in this study for running the models to predict the CS and STS of RCA-based concrete were taken from the published literature and are available in Appendix A [27]. The anaconda navigator software was used in this research and incorporated Python coding to run the models for forecasting the results. The excel file with relevant input and output data was uploaded to the software, which runs the model as per the data available in the file. The outcome from the model was then imported for graphical representation. The running of the models comprised nine input parameters (cement, water, fine aggregate, natural CA, RCA, superplasticizers, maximum size of RCA, density of RCA, water absorption of RCA) and two output parameters (CS and STS). The relative frequency distribution of the nine variables can be seen in Figure 1. The relevant references regarding the application of various ML approaches are listed in Table 1. The descriptive statistical analysis for input parameters is illustrated in Table 2, indicating the various mathematical description and ranges of input parameters. In addition, the methodology of the research approach is presented via flowchart, as depicted in Figure 2, which represents the information of the stepwise adopted procedure of the study. The first phase indicates the information of the data obtained, and then the analysis took place using machine learning algorithms, while result explanation, comparison, and evaluation are presented in the next step of the flowchart.

## 3. Supervised Machine Learning Algorithms

### 3.1. Decision Tree Algorithm

The DT algorithm is a subset of the supervised machine learning (ML) technique known as individual supervised machine learning (ISML). It is applicable to classification and regression problems. This approach aims to generate a model that can forecast the targeted variable, for which it uses the representation of a tree to solve the problem. In machine learning, the classification process has two steps, the learning and forecasting steps. The learning step belongs to the development of the model based on the given data set, while, in the prediction step, said model is then used to foretell the response of the data. A decision tree is a well-known and effective classification technique that is simple to comprehend and apply. Sub-node creation improves the homogeneity of specific sub-nodes. There are several important terminologies associated with the decision tree. These include root nodes, which indicate the overall population of the sets; splitting, which refers to the process of dividing the nodes; decision nodes, which refers to the process of splitting sub-nodes into further sub-nodes; leaf nodes, which are the type of nodes that do not split; and pruning, which refers to the process of removing sub-nodes.

### 3.2. AdaBoost Algorithm

The AdaBoost regressor is a supervised ML technique that uses an ensemble approach. It is also known as adaptive boosting because the weights are re-assigned to each instance, with greater weights going to instances that were mistakenly identified. Boosting techniques are commonly used in supervised learning to reduce bias and variation. These ensemble algorithms are used to improve the performance of the weak learner. During the training phase for the input data, it uses an endless number of decision trees. The recorded data that are incorrectly categorized throughout the initial model are given a high priority while developing the initial decision tree/model. These are the only data entries that are utilized as the input for a different model. The preceding technique will be repeated until the desired number of basic learners has been reached. When it comes to binary classification problems, the AdaBoost regressor outperforms the competition in terms of improving decision tree performance. It is also used to boost the efficiency of other machine learning methods. When used with a slow student, it is quite beneficial. The use of these ensemble methods is most common in civil engineering, especially when it comes to predicting the mechanical properties of different types of concrete.

## 4. Result and Their Analyses

### 4.1. Statistical Analysis

The result obtained from the statistical analyses indicated that the relationship between the actual and predicted outcomes (CS and STS) from the individual and ensemble ML algorithms, along with the distribution of errors, is explained as follows.

#### 4.1.1. Compressive Strength Result Using Decision Tree

**Figure 3:**
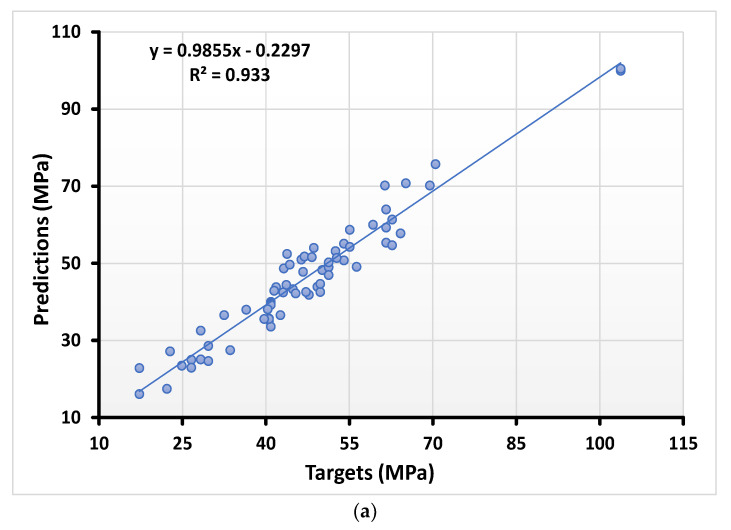

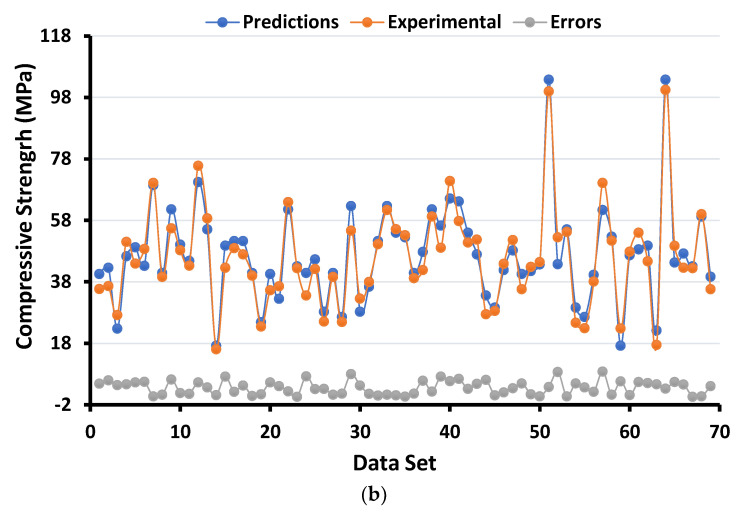
Numerical analyses representing the relationship between the predicted variables and targeted variables (**a**) along with their error distribution (**b**) for compressive strength using DT.

The relationship between the actual and predicted result of compressive strength for the decision tree algorithm can be seen in Figure 3a, along with the distribution of the errors shown in Figure 3b. The errors distribution for DT gives the maximum, minimum, and average values equal to 8.82 MPa, 0.58 MPa, and 3.58 MPa, respectively. However, 11.59% of the error data lie between 0 and 1 MPa, and 50.72% of the data lie between 2 MPa and 6 MPa. In addition, only 8.69% of the data lie above 7 MPa.

#### 4.1.2. Splitting Tensile Strength Result Using Decision Tree

**Figure 4:**
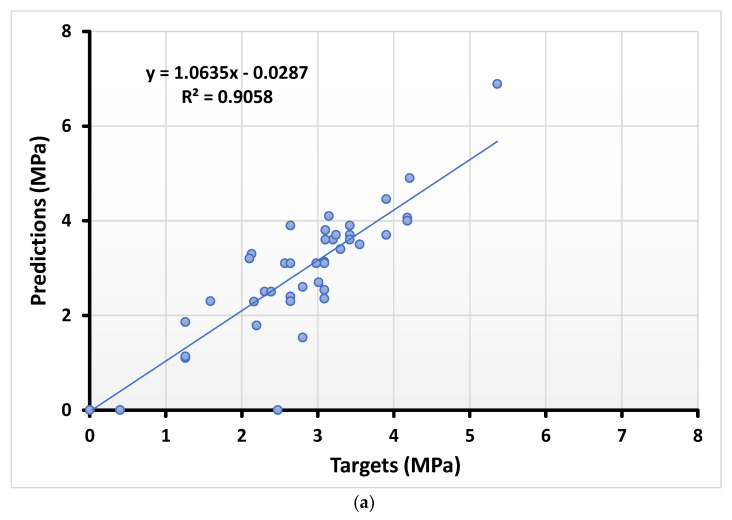

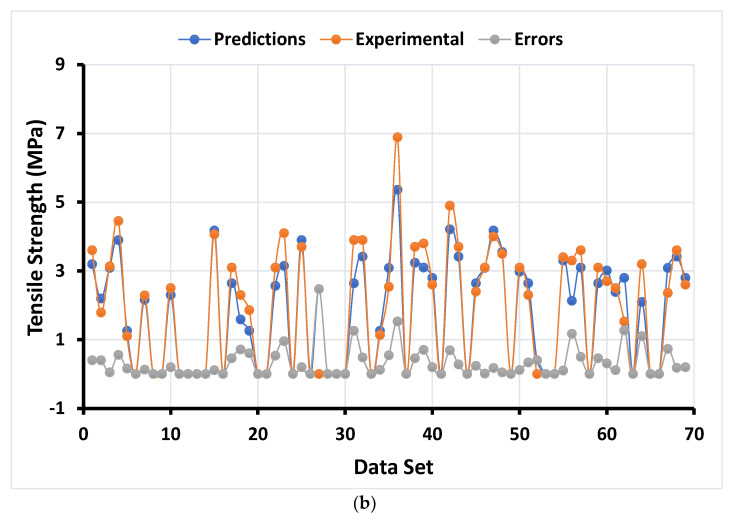
Numerical analyses representing the relationship between the predicted variables and targeted variables (**a**) along with their error distribution (**b**) for splitting tensile strength using DT.

The relation of the actual and predicted outcome of splitting tensile strength using the DT approach in depicted in Figure 4a along with its error distribution depicted in Figure 4b. The error distribution indicates the higher, lower, and average values equal to 2.47 MPa, 0, and 0.31 MPa, respectively. In contrast, 42.02% of the error data lie between 0 and 0.1 MPa, while 34.78% of the data lie between 0.1 MPa and 0.5 MPa. However, only 8.69% of the error data were reported as above 1 MPa.

#### 4.1.3. Compressive Strength Result with AdaBoost Regressor

**Figure 5:**
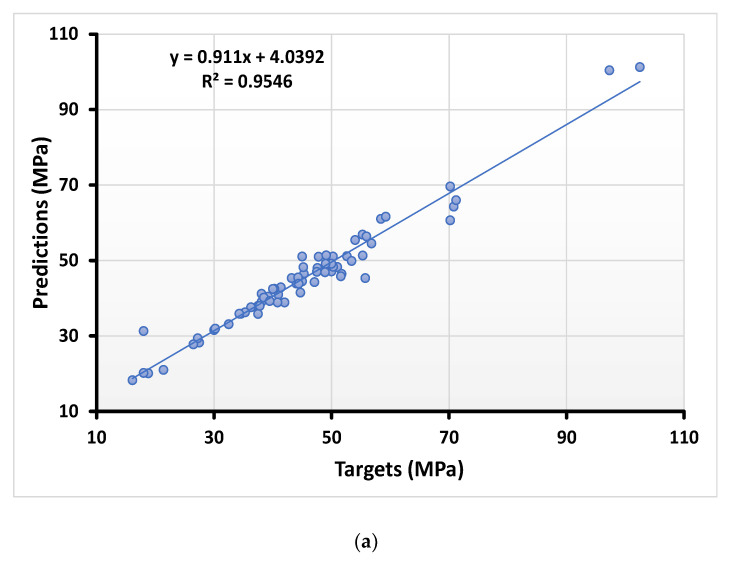

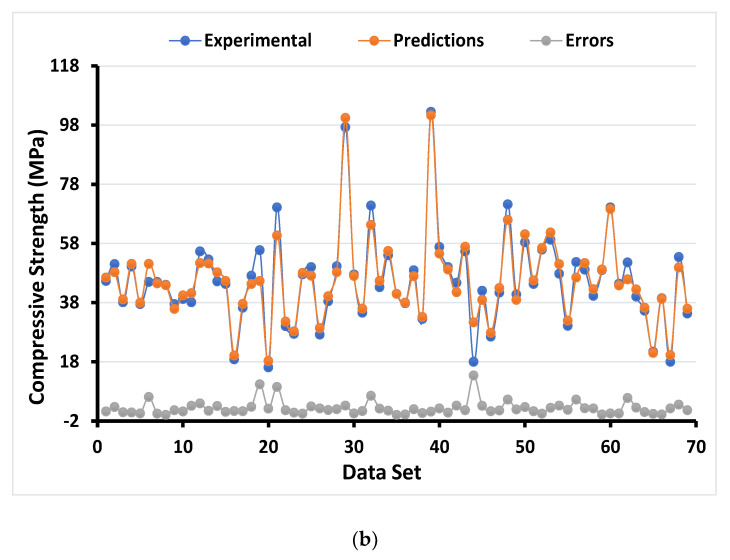
Numerical analyses representing the relationship between the predicted variables and targeted variables (**a**) along with their error distribution (**b**) for compressive strength using the AdaBoost regressor.

AdaBoost regressor gives strong relation between the real and anticipated output, as shown in Figure 5a, while the distribution of the error’s value can be seen in Figure 5b. It shows the maximum, lower, and average values of the error data equal to 13 MPa, 0.06 MPa, and 2.33 MPa, respectively. Additionally, 26.08% of the error data were reported between 0 and 1 MPa, while 34.78% of the data lie between 2 MPa and 6 MPa. However, 4.34% of the error data were reported to be above 7 MPa.

#### 4.1.4. Splitting Tensile Strength with AdaBoost Regressor

**Figure 6:**
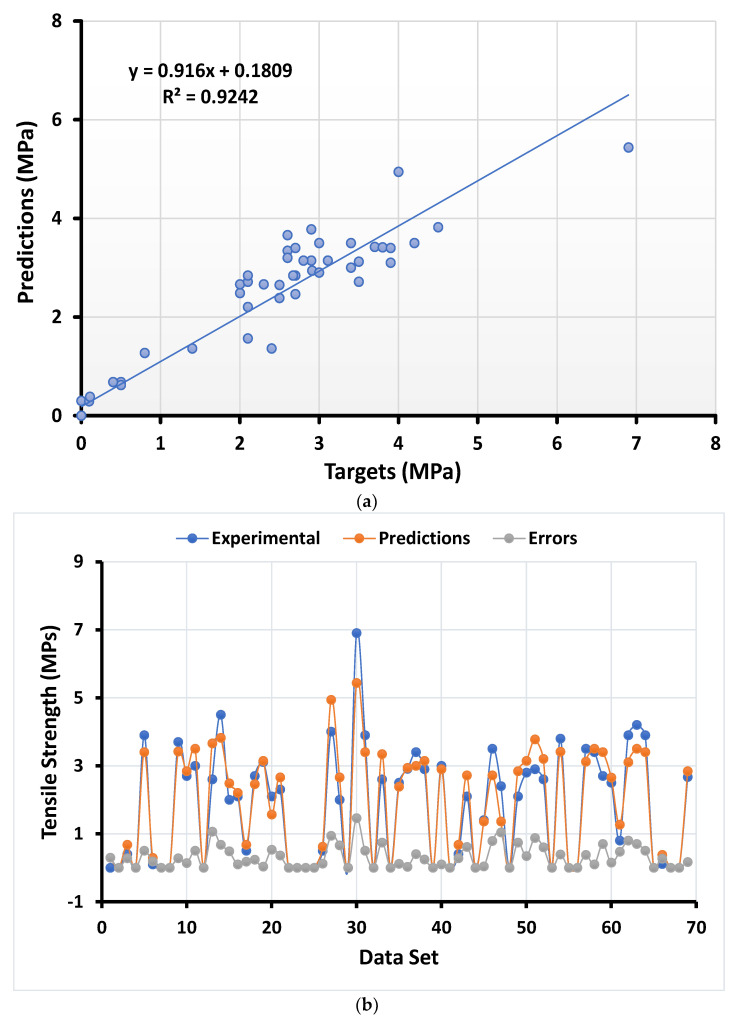
Numerical analyses representing the relationship between the predicted variables and targeted variables (**a**) along with their error distribution (**b**) for splitting tensile strength using the AdaBoost regressor.

The statistical result of splitting tensile strength using the AdaBoost regressor also shows strong relations with less variance among the experimental results obtained from the model, as depicted in Figure 6a. The distribution of the errors obtained from the application of the AdaBoost regressor can be seen in Figure 6b. The error distribution shows the maximum, minimum, and average values equal to 1.46 MPa, 0, and 0.30 MPa, respectively. However, 36.26% of the error data lie between 0 and 0.1 MPa, while 34.78% of the data lie between 0.1 MPa and 0.5 MPa. In addition, only 4.34% of the errors data lie above 1 MPa.

### 4.2. K-Fold Cross-Validation and Statistical Checks

This process is normally adopted to check the authentic execution of the models. The authentic performance of the employed models is being verified from the k-fold cross-validation process. In this method, the available data set is arranged randomly and split up into ten groups. A total of 60% of the dataset from total data points were used to train the model, 30% of the dataset were used to test the model, and 10% of the data were used for validation purposes. The process takes place in such a way that nine groups from ten are assigned for training the models, while the remaining one is for validation of the models. The said process was again repeated ten times to obtain the suitable average value. The K-fold cross-validation process also confirms the performance accuracy of the models. The statistical checks to confirm the accuracy level of the model’s prediction were also employed using the equations illustrated below from (1)–(5)

(1) $$\mathrm{RMSE}=\sqrt{\frac{\sum_{i=1}^{n}\;{\left(ex_{i}-mo_{i}\right)}^{2}}{n}}$$

(2) $$\mathrm{MAE}=\frac{\sum_{i=1}^{n}|ex_{i}-mo_{i}|}{n}$$

(3) $$\mathrm{RSE}=\frac{\sum_{i=1}^{n}{\left(mo_{i-}ex_{i}\right)}^{2}}{\sum_{i=1}^{n}{\left(\bar{ex}-ex_{i}\right)}^{2}}$$

(4) $$\mathrm{RRMSE}=\frac{1}{e}\sqrt{\frac{\sum_{i=1}^{n}{\left(ex_{i}-mo_{i}\right)}^{2}}{n}}$$

(5) $$R=\frac{\sum_{i=1}^{n}\left(ex_{i}-{\bar{ex}}_{i}\right)\left(mo_{i}-{\bar{mo}}_{i}\right)}{\sqrt{\sum_{i=1}^{n}{\left(ex_{i}-{\bar{ex}}_{i}\right)}^{2}\sum_{i=1}^{n}{\left(mo_{i}-{\bar{mo}}_{i}\right)}^{2}}}$$

where,

$ex_{i}$ = experimental value,

$mo_{i}$ = predicted value,

${\bar{ex}}_{i}$ = mean experimental value,

${\bar{mo}}_{i}$ = mean predicted value obtained by the model,

*n* = number of samples.

**Figure 7:**
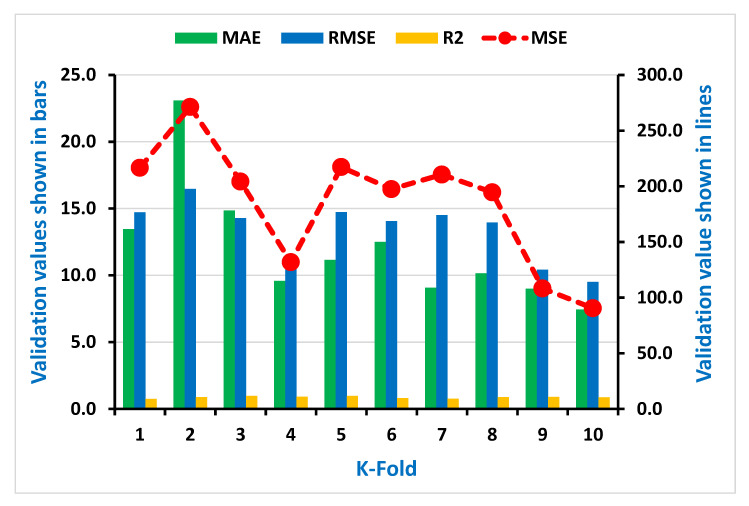
Representation of statistical information of k-fold cross-validation using DT for compressive strength.

**Figure 8:**
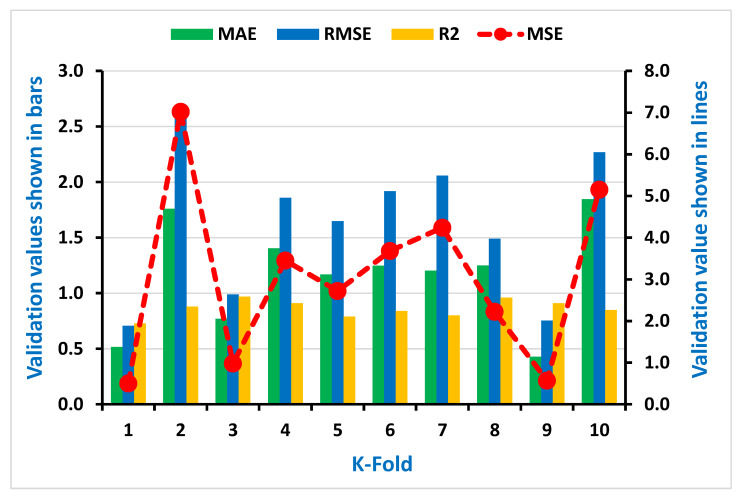
Representation of statistical information of k-fold cross-validation using DT for splitting tensile strength.

**Figure 9:**
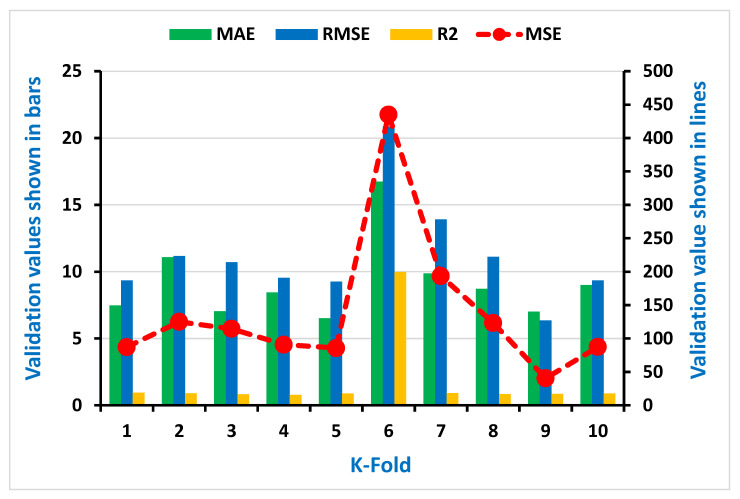
Representation of statistical information of k-fold cross-validation using AdaBoost for Compressive strength.

**Figure 10:**
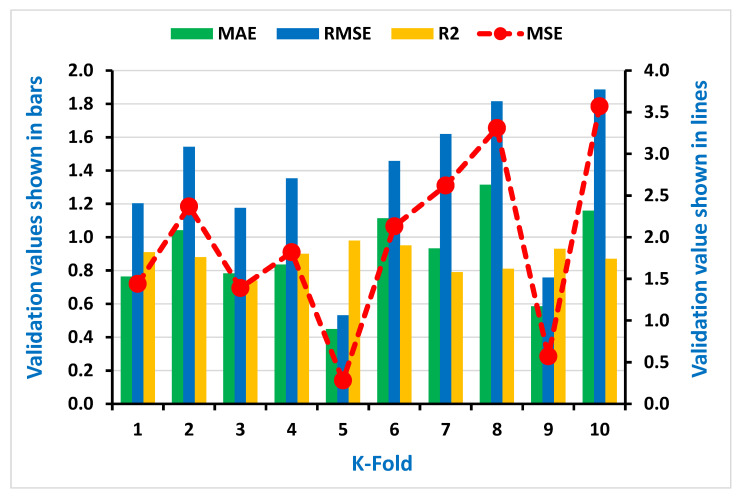
Representation of statistical information of k-fold cross-validation using AdaBoost for Splitting tensile strength.

As seen in Figure 7, Figure 8, Figure 9 and Figure 10, the coefficient correlation (R^2^), mean square error (MSE), mean absolute error (MAE), and root mean square error (RMSE) were used to evaluate the k-fold cross-validation of each employed model against its output. The variation was also noticed in the outcomes of both ML algorithms used (DT and AdaBoost). The lower the number of errors in the AdaBoost model, the higher the coefficient correlation (R^2^) value, indicating a higher accuracy level than the decision tree. The information obtained from the analysis for both CS and STS used for k-fold cross-validation is listed in Table 3 and Table 4, respectively.

Additionally, the information of statistical checks in the form of MAE, MSE, and RMSE were assessed for both CS and STS and can be seen in the Table 5 and Table 6, respectively. The lesser error shows a higher coefficient correlation value (R^2^).

## 5. Sensitivity Analyses

**Figure 11:**
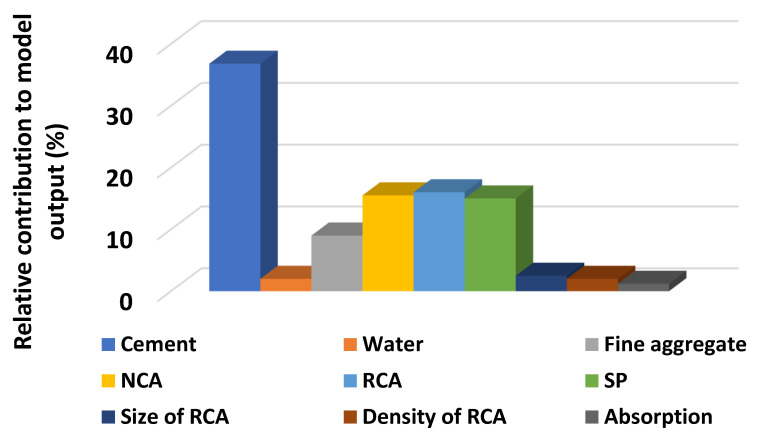
Contributing level of the input variables towards the prediction of output for CS.

**Figure 12:**
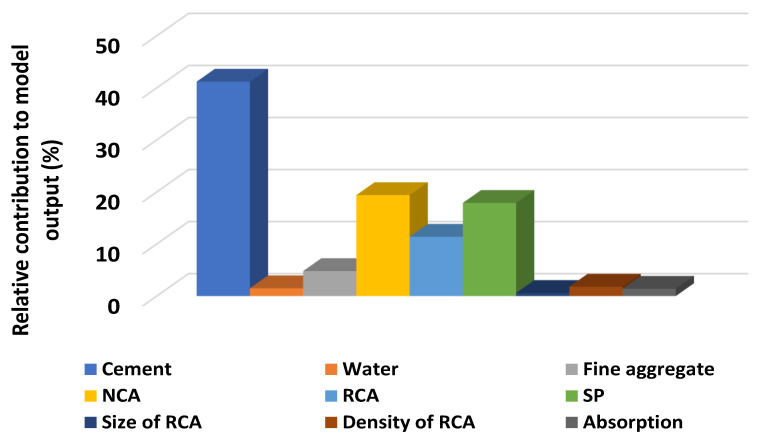
Contributing level of the input variables towards the prediction of output for STS.

The input variables have a remarkable effect on the execution of the model’s outcome. The sensitivity analyses were done to investigate the effect of each variable on the anticipation of both STS and CS, as depicted in Figure 5. The cement significantly contributed (36.8%) towards the prediction of CS, while other parameters contributed the least towards the forecasting of concrete CS containing RCA, as shown in Figure 11. However, the contribution of parameters for predicting the STS can be seen in Figure 12. The significant contributions for the prediction of the STS of concrete were cement (41.2%) and natural coarse aggregate (NCA) (19%), while superplasticizers and RCA were the next highest contributors for the prediction of outcomes. The equations mentioned below were used to calculate the contribution of each parameter towards the model’s outcome.

(6) $$N_{i}=f_{max}\left(x_{i}\right)-f_{min}\left(x_{i}\right)$$

(7) $$S_{i}=\frac{N_{i}}{\sum_{j-i}^{n}N_{j}}\;$$

where—*f_min_* (*x_i_*) and *f_max_* (*x_i_*) are the lower and higher of the estimated output over the *i*th output.

## 6. Discussion

As demonstrated by the data, the ML-based strategy for forecasting the mechanical characteristics of concrete is clearly better than traditional mechanics-based methods. The advantages are as follows: (1) ML does not at all require complex mechanics/theoretical equations but instead finds the mapping between the input and output utilizing numerical and/or computer knowledge of science, making it very accessible to the readers; (2) Unlike most empirical models, which typically consider a limited number of variables when deriving the formula, ML can consider an infinite number of variables; (3) Meanwhile, inherent uncertainties [55] can be incorporated into the training process; and (4) The precision, reliability, and robustness of machine learning-based models are significantly higher than those of traditional models: they can provide objective and accurate results in a matter of seconds.

The research approach of this study was to predict the mechanical properties (CS and STS) of concrete containing recycled coarse aggregates (RCA) via supervised machine learning algorithms. The anaconda navigator software was used to incorporate the Python coding for each employed machine learning algorithm. An excel file having a relevant database was used in the software, which allowed it to show the output results in the form of R^2^, MAE, MSE, and RMSE. The AdaBoost technique performs well, as proven by the coefficient correlation (R^2^) value of 0.95 for CS prediction and 0.92 for STS prediction., Feng et al. [56] Additionally, AdaBoost was used to classify failure modes, yielding an accuracy of 0.96, and to determine the bearing capacity of reinforced concrete, yielding an R^2^ value of 0.98. However, the value of R^2^ for DT in predicting the CS was 0.93, and in forecasting, the STS was equal to 0.90. In comparison, Ahmad et al. [57] also employed DT to predict the CS of geopolymer concrete, which shows a reasonable and almost similar value of R^2^ equal to 0.90 for its outcome. The higher value of R^2^ (0.95 for compressive and 0.92 for STS) for AdaBoost indicates the high performance towards the prediction of the outcomes as compared to the R^2^ value for DT (0.93 for compressive and 0.90 for STS). The lesser values of each error (MAE, MSE, RMSE) for AdaBoost also confirm the model’s better accuracy level as opposed to the errors values for the DT. In addition, the sensitivity analysis describes the contribution level of each parameter used to run the model for predicting the mechanical properties of concrete containing recycled coarse aggregates. Cement and natural coarse aggregate (NCA) contributed significantly, up to 41.2% and 19%, respectively, while superplasticizers and RCA were the next highest contributors for the prediction of outcomes. It was noted that the accuracy level of the ensemble machine learning approach (AdaBoost) was higher than the individual machine learning technique (DT).

## 7. Conclusions and Future Recommendations

This research describes the application of both individual and ensemble ML algorithms to forecast the mechanical properties such as compressive strength (CS) and splitting tensile strength (STS) of concrete having recycled coarse aggregate (RCA). The decision tree (DT) and AdaBoost approaches were incorporated for prediction purposes. The input variables were analyzed by indicating their relative frequency distribution. The Python coding was used in the Spyder (Anaconda software) to run the required models for further investigation. The statistical checks in the form of various errors (MAE, RMSE, MSE) were evaluated to confirm the accuracy of each employed model. However, the k-fold cross-validation method was also included in the study for the confirmation of the model’s accuracy. In addition, the contribution of each input variable was investigated via sensitivity analysis. The following conclusions and future recommendations can be drawn from the study.

- The ensemble machine learning algorithm (AdaBoost) shows a better response with less variance towards the prediction of both the CS and splitting tensile strength of RCA-based concrete.
- The AdaBoost regressor gives the values of coefficient correlation (R^2^) for CS and STS of concrete equal to 0.95 and 0.92, respectively, as opposed to the values of R^2^ for DT equal to 0.93 (CS) and 0.90 (STS).
- The higher values of R^2^ for the AdaBoost regressor towards the prediction of both CS and STS indicate the high accuracy of the model.
- From the statistical checks, the lesser value of the errors (MAE, MSE, RMSE) also indicates high performance for the AdaBoost approach compared to the DT algorithm.
- The K-fold cross-validation method also confirms the high accuracy level of the AdaBoost algorithm.
- Sensitivity analysis reveals that the cement contributed effectively (32%) as compared to other parameters towards the forecasting of the CS of RCA-based concrete, while the superplasticizers were the higher contributor towards the prediction of the STS of concrete containing RCA.

In conclusion, this study was based on the application of supervised machine learning (ML) algorithms to foretell the two parameters (CS and STS) of concrete having recycled coarse aggregate (RCA). It also gives an idea of the importance of multiple aspects like the input variables, the number of data points for running the models, and the types of ML approaches to be used for high accuracy of the outcomes. The algorithms employed in this study show a strong relationship between the actual and predicted output. The importance of these approaches in civil engineering is indicated by their high accuracy level among the real and forecasted results. The supervised ML approaches are gaining more popularity, as their application gives high accuracy results/outcomes and minimizes the physical approach of the practical work and total cost of the project. Additionally, it is essential to incorporate laboratory work to compare machine learning approaches’ findings to better understand their effectiveness. Additionally, the data points, type of material used, size of specimens, environmental conditions, curing conditions, loading rate, and increase in the input parameters can be modified or added to study and compare the results of various machine learning algorithms. Moreover, various ML techniques such as artificial neural networks (ANN), support vector machines (SVM), and boosting can be included to evaluate their performance.

## Figures and Tables

**Table 1:** Various predicted properties with the application of ML approaches.

Sr. No	Algorithm Used	Notation	Data Points	Prediction Properties	Year	Material Used	References
1.	Support vector machine	SVM	144	CS	2021	Fly ash (FA)	[28]
2.	Gene expression programming	GEP	303	Column’s bearing capacity	2019	_	[29]
3.	Data Envelopment Analysis	DEA	114	Fresh and harden properties of concrete	2021	FA	[30]
4.	Gene expression programming, Artificial neural network, Decision tree	GEP, ANN, DT	642	Surface Chloride Concentration	2021	FA	[31]
5.	Support vector machine	SVM	-	CS	2020	FA	[32]
6.	Support vector machine	SVM	115	Fresh properties of concrete CS	2020	FA	[33]
7.	Gene Expression Programming	GEP	351	CS	2020	Ground Granulated Blast Furnace Slag	[34]
8.	Gene Expression Programming	GEP	54	CS	2019	NZ (Natural Zeolite)	[34]
9.	Gene expression programming	GEP	357	CS	2020	-	[35]
10.	Random forest and gene expression programming	RF and GEP	357	CS	2020	-	[36]
11.	Artificial neuron network	ANN	205	CS	2019	Fly ash GGBFS Rice husk ash	[37]
12.	Intelligent rule-based enhanced multiclass support vector machine and fuzzy rules	IREMSVM-FR with RSM	114	CS	2019	Fly ash	[38]
13.	Random forest	RF	131	CS	2019	Fly ash GGBFS FA	[39]
14.	Multivariate adaptive regression spline	M5 MARS	114	CS Slump test L-box test V-funnel test	2018	FA	[40]
15.	Random Kitchen Sink Algorithm	RKSA	40	V-funnel test J-ring test Slump test CS	2018	FA	[41]
16.	Adaptive neuro fuzzy inference system	ANFIS	55	CS	2018	-	[42]
17.	Artificial neuron network	ANN	114	CS	2017	FA	[43]
18.	Artificial neuron network	ANN	69	CS	2017	FA	[44]
19	Individual and ensemble algorithm	GEP, DT, Bagging	270	CS	2021	FA	[45]
20.	Individual with ensemble modeling	ANN, bagging, boosting	1030	CS	2021	FA	[46]
21.	Multivariate	MV	21	CS	2020	Crumb rubber with SF	[47]
22.	Gene expression programming	GEP	277	Axial capacity	2020	-	[48]
23.	Adaptive neuro fuzzy inference system	ANFIS with ANN	7	CS	2020	Palm oil fuel ash	[49]
24.	Response Surface Method, Gene expression programming	RSM, GEP	108	CS	2020	Steel Fibers	[50]
25.	Artificial neural network	ANN	60	CS	2021	Ceramic waste powder	[51]
27.	Decision tree, Artificial neural network, Bagging, Gradient boosting	DT, ANN, BR, GB	207	CS	2021	FA	[52]
28.	Gene expression programming, Artificial neural network, Decision tree	GEP, ANN, DT	98	CS	2021	FA	[53]
29.	Individual and Ensemble techniques	BR, GEP, DT	1030	CS	2021	FA	[54]

**Table 2:** Details of the descriptive analysis.

Statistics	Water	Cement	FA	NCA	RCA	SP	RCA Size	RCA Density	Absorption
Mean	184.62	386.86	681.89	398.07	650.74	1.32	19.76	2231.06	4.80
Standard Error	1.39	4.43	11.07	19.99	20.37	0.11	0.22	31.32	0.12
Median	180.00	380.00	698.00	471.00	552.00	0.00	20.00	2362.50	4.90
Mode	220.00	380.00	693.00	0.00	138.00	0.00	20.00	2320.00	5.30
Standard Deviation	25.84	82.16	205.28	370.71	377.73	2.05	4.02	580.95	2.26
Sample Variance	667.47	6750.28	42,141.11	137,424.94	142,682.56	4.21	16.16	337,504.80	5.12
Minimum	117.6	158	0	0.00	52	0.	10	0	0
Maximum	271.	600	1010	1448	1778	7.8	32	2661	10.9
Sum	63,510.69	133,081.00	234,568	136,937	223,853	455.5	6796	767,484	1652.8
Count	344.00	344.00	344	344	344	344	344	344	344

**Table 3:** Result of k-fold cross-validation for compressive strength.

AdaBoost					Decision Tree				
K-Fold	MAE	MSE	RMSE	R^2^	K-Fold	MAE	MSE	RMSE	R^2^
1	7.49	87.20	9.34	0.95	1	13.45	216.60	14.72	0.76
2	11.08	124.99	11.18	0.91	2	23.10	271.26	16.47	0.89
3	7.04	114.49	10.70	0.83	3	14.85	204.20	14.29	0.97
4	8.45	90.82	9.53	0.79	4	9.59	131.79	11.48	0.92
5	6.52	85.74	9.26	0.90	5	11.17	217.26	14.74	0.98
6	16.74	435.13	20.86	9.98	6	12.51	197.40	14.05	0.82
7	9.88	193.48	13.91	0.93	7	9.07	210.54	14.51	0.77
8	8.72	123.43	11.11	0.85	8	10.15	194.60	13.95	0.88
9	7.02	40.44	6.36	0.87	9	9.00	108.16	10.42	0.90
10	9.00	87.42	9.35	0.90	10	7.44	90.44	9.51	0.87

**Table 4:** Result of k-fold cross-validation for splitting tensile strength.

AdaBoost					Decision Tree				
K-Fold	MAE	MSE	RMSE	R^2^	K-Fold	MAE	MSE	RMSE	R^2^
1	0.76	1.44	1.20	0.91	1	0.52	0.50	0.71	0.73
2	1.04	2.37	1.54	0.88	2	1.76	7.02	2.65	0.88
3	0.78	1.39	1.18	0.74	3	0.77	0.98	0.99	0.97
4	0.84	1.82	1.35	0.90	4	1.41	3.45	1.86	0.91
5	0.45	0.28	0.53	0.98	5	1.17	2.72	1.65	0.79
6	1.11	2.13	1.46	0.95	6	1.25	3.68	1.92	0.84
7	0.93	2.62	1.62	0.79	7	1.20	4.24	2.06	0.80
8	1.31	3.31	1.82	0.81	8	1.25	2.22	1.49	0.96
9	0.59	0.57	0.76	0.93	9	0.43	0.57	0.75	0.91
10	1.16	3.57	1.89	0.87	10	1.85	5.15	2.27	0.85

**Table 5:** Statistical checks for compressive strength.

Algorithms Used	MAE (MPa)	MSE (MPa)	RMSE (MPa)
Decision tree (DT)	3.58	11.02	3.32
AdaBoost	2.33	7.8	2.79

**Table 6:** Statistical checks for splitting tensile strength.

Algorithm Used	MAE (MPa)	MSE (MPa)	RMSE (MPa)
Decision tree (DT)	0.31	0.29	0.54
AdaBoost	0.30	0.20	0.45

## Data Availability

Not applicable.

## Appendix A

**Table:**

Water (kg/m^3^).	Cement (kg/m^3^)	Sand (kg/m^3^)	NCA (kg/m^3^)	RCA (kg/m^3^)	SP (kg/m^3^)	RCA Size (kg/m^3^)	Density (kg/m^3^)	Absorption (%)	CS (MPa)	STS (MPa)
165	370	650	850.5	364.5	2.22	20	2400	4.9	50.6	-
165	370	650	607.5	607.5	2.22	20	2400	4.9	50.8	-
165	370	650	-	1215	2.22	20	2400	4.9	50.2	-
165	460	575	850.5	364.5	2.22	20	2400	4.9	60.8	-
165	460	575	607.5	607.5	2.22	20	2400	4.9	61.2	-
165	460	575	-	1215	2.22	20	2400	4.9	60.2	-
165	560	495	850.5	364.5	2.59	20	2400	4.9	70.2	-
165	560	495	607.5	607.5	2.59	20	2400	4.9	70.8	-
165	560	495	-	1215	2.59	20	2400	4.9	70	-
180	500	486.6	-	1135.4	-	16	-	-	44.5	4
180	500	-	-	1574.3	-	16	-	-	38.7	3.5
180	500	486.6	-	1135.4	-	16	-	-	46.1	3.4
180	500	-	-	1574.3	-	16	-	-	42.4	3.2
180	500	486.6	-	1135.4	-	16	-	-	52.5	4
180	500	-	-	1574.3	-	16	-	-	50.7	3.6
180	500	486.6	-	1135.4	-	16	-	-	45.2	3.5
180	500	-	-	1574.3	-	16	-	-	42	3.2
180	500	486.6	-	1135.4	-	16	-	-	49.6	3.8
180	500	-	-	1574.3	-	16	-	-	45.1	3.5
180	500	509.6	-	1135.4	-	16	-	-	54.4	4
180	500	-	-	1574.3	-	16	-	-	48.2	3.8
207.6	400	662	863	153	-	20	2410	5.8	38.1	3.7
207.6	400	662	697	298	-	20	2410	5.8	37	3.6
207.6	400	662	383	573	-	20	2410	5.8	35.8	3.4
207.6	400	662	-	903	-	20	2410	5.8	34.5	3.3
217	353	660	861	209	-	20	2330	6.3	44.9	-
229	353	647	527	513	-	20	2330	6.3	44.7	-
241	353	625	-	993	-	20	2330	6.3	46.8	-
230	353	661	853	202	-	20	2330	6.3	43.2	-
247	353	647	524	496	-	20	2330	6.3	39.7	-
271	353	625	-	959	-	20	2330	6.3	43.3	-
206	353	661	864	216	-	20	2330	6.3	43	-
207	353	649	531	531	-	20	2330	6.3	38.1	-
165	300	765	905	267	4.98	25	2430	4.4	42	3
165	318	739	608	537	6.042	25	2430	4.4	41	3.2
162	325	683	-	1123	6.175	25	2430	4.4	40	3.2
160.6	380	598	1182	52	4.9	20	2165	6.8	62.2	-
165.4	380	529	1175	103	4.9	20	2165	6.8	58.4	-
170.2	380	460	1168	154	4.9	20	2165	6.8	61.3	3.7
175.6	380	327	1162	254	4.9	20	2165	6.8	60.8	-
180.9	380	-	1162	509	4.9	20	2165	6.8	61	3
225	410	642	840	204	-	20	2570	3.5	45.3	-
225	410	642	524	506	-	20	2570	3.5	42.5	-
225	410	642	210	814	-	20	2570	3.5	39.2	-
225	410	642	-	1017	-	20	2570	3.5	37.1	-
180	400	708	886	215	-	20	2570	3.5	62.4	3.2
180	400	708	554	538	-	20	2570	3.5	55.8	3
180	400	708	-	1075	-	20	2570	3.5	42	2.8
225	410	642	840	204	-	20	2570	3.5	45.3	3.2
225	410	642	524	506	-	20	2570	3.5	42.5	3.2
225	410	642	-	1017	-	20	2570	3.5	38.1	3.1
234	360	705	-	1100	-	19	2390	4.4	22.1	-
190	380	705	-	1100	-	19	2390	4.4	25.1	-
192	400	705	-	1100	-	19	2390	4.4	27.2	-
181	420	705	-	1100	-	19	2390	4.4	28.7	-
184	460	705	-	1100	-	19	2390	4.4	29.5	-
178	264	835	-	1030	-	30	2520	3.8	18	-
174	262	830	-	1020	-	30	2510	3.9	15.4	-
148	427	760	-	1000	4.2	30	2520	3.8	36.4	-
153	423	755	-	990	4.1	30	2510	3.9	35.7	-
152	443	855	-	885	3.9	30	2520	3.8	44.4	-
225	410	642	840	204	-	20	2580	3.5	45.3	3.6
225	410	642	524	506	-	20	2580	3.5	42.5	3.4
225	410	642	-	1017	-	20	2580	3.5	38.1	3.3
205	410	662	865	210	-	20	2580	3.5	51.7	3.6
205	410	662	541	525	-	20	2580	3.5	47.1	3.6
205	410	662	-	1049	-	20	2580	3.5	43.4	3.4
180	400	708	886	215	5.6	20	2580	3.5	62.4	3.7
180	400	708	554	538	5.6	20	2580	3.5	56.8	3.7
180	400	708	-	1075	5.6	20	2580	3.5	52.1	3.5
160	400	729	912	221	7.8	20	2580	3.5	69.6	4.1
160	400	729	570	554	7.8	20	2580	3.5	65.3	4
160	400	729	-	1107	7.8	20	2580	3.5	58.5	3.8
175	350	730	711	297	1.68	25	2530	1.9	36.7	4.2
175	350	730	508	494	1.68	25	2530	1.9	38	4
175	350	730	-	989	1.68	25	2530	1.9	36	3.8
175	350	730	711	282	1.68	25	-	-	32.6	3.6
175	350	730	508	469	1.68	25	2400	6.2	30.4	3.4
175	350	730	-	938	1.68	25	2400	6.2	29.5	3.2
190	380	744.45	756.97	189.24	2.66	20	2338	5.2	47.4	-
190	380	709.54	471.13	471.12	2.66	20	2338	5.2	47.3	-
190	380	714.56	-	874.04	5.32	20	2338	5.2	54.8	-
140	350	732	519	556	4.2	12	2420	6.8	43.3	-
153	340	723	512	549	3.4	12	2400	6.8	39.6	-
165	330	715	507	543	2.64	12	2400	6.8	38.1	-
176	320	708	502	537	1.92	12	2400	6.8	34.5	-
186	310	702	497	533	1.24	12	2400	6.8	31.6	-
140	350	732	553	523	4.2	22	2420	8.8	46.1	-
153	340	723	547	517	3.4	22	2420	8.8	45.8	-
165	330	715	541	511	2.64	22	2420	8.8	39.9	-
176	320	708	535	506	1.92	22	2420	8.8	36.3	-
186	310	702	531	501	1.24	22	2420	8.8	34.7	-
186	372	617.65	1030.22	257.56	-	20	2400	-	27.2	3.1
186	372	617.65	772.67	515.55	-	20	2400	-	26.5	2.8
186	372	617.65	515.11	772.67	-	20	2400	-	25.4	2.7
186	372	617.65	257.56	1030.22	-	20	2400	-	25.1	2.2
186	372	494.12	128.78	123.53	-	20	2630	-	26.4	2.6
186	372	370.59	128.78	247.06	-	20	2630	-	25.9	2.5
186	372	247.06	128.78	370.59	-	20	2630	-	23.5	2.3
186	372	123.53	128.78	494.12	-	20	2630	-	15.4	2
200	270	750	675	200	1.08	19	2440	5.8	18.5	1.9
210	270	750	450	400	1.35	19	2440	5.8	18	1.9
220	270	750	225	600	1.62	19	2440	5.8	16.5	1.4
165	370	865	760	230	1.48	19	2440	5.8	33	3.1
165	370	865	505	455	1.85	19	2440	5.8	34.5	3.1
165	370	865	250	680	2.59	19	2440	5.8	34	2.9
178.5	275	938.05	723.07	180.77	1.925	16	2400	5	31.7	2.4
178.5	275	962.73	423.77	423.77	1.925	16	2400	5	32.4	2.5
178.5	275	1005.18	-	756.46	1.925	16	2400	5	30.1	2.6
190	380	794.31	750.04	187.57	2.66	16	2400	5	43.7	3.1
190	380	811.37	443.71	443.71	2.66	16	2400	5	37.5	2.9
190	380	838.29	-	807.97	2.66	16	2400	5	40.5	2.9
151	335	630	414	720	1.266	19	2420	5.4	41.4	-
156	349	888	-	792	1.67616	19	2420	5.4	43.9	-
161	358	645	281	813	1.3584	19	2500	3.3	44.8	-
156	349	857	-	867	1.2564	19	2500	3.3	45.9	-
172.43	401	574	911	303	0.2005	20	2661	1.9	47	2.3
172.43	401	574	585	585	0.70175	20	2602	2.6	46	2.1
172.43	401	574	-	1119	0.90225	20	2510	3.9	42.5	2
190.8	424	770	-	980	-	19	2490	4.8	41	-
192.5	350	800	-	1015	-	19	2490	4.8	33.3	-
191.75	295	814	-	1039	-	19	2490	4.8	24.8	-
150	250	762	858	286	4.375	19	-	-	26.7	2.2
150	250	753	564	564	4.375	19	-	-	21.5	1.8
150	250	743	279	836	4.375	19	-	-	21.4	1.4
150	250	734	-	1100	4.375	19	-	-	20	1.2
180	400	685	770	257	3	19	-	-	38.3	3.1
180	400	676	507	507	3	19	-	-	37	2.7
180	400	667	250	751	3	19	-	-	35	2.5
180	400	659	-	988	3	19	-	-	33.3	2.1
175	325	-	-	1762	3.45	32	2263	6	33.2	-
222	350	-	-	1778	4.5	32	2283	4.2	35.6	-
221	350	-	-	1771	4.5	32	2292	4.3	34.6	-
195	325	-	-	1710	3.25	32	2301	5	37.3	-
123	300	-	192	1728	3	32	2609	1.5	45.4	-
144	325	-	768	1152	3.25	32	2518	2.7	54.3	-
123	325	-	754.4	1131.6	3.25	32	2584	1.6	54.4	-
132	300	-	1448.25	482.75	3	32	2594	1.6	53.4	-
180	275	625	882	378	-	20	2340	5.3	20	-
180	295	595	635	635	-	20	2340	5.3	19	-
180	310	610	-	1240	-	20	2340	5.3	18	-
180	330	585	872	373	-	20	2340	5.3	23	-
180	355	560	623	623	-	20	2340	5.3	24	-
180	372	536	-	1252	-	20	2340	5.3	21	-
180	355	560	872	373	-	20	2340	5.3	25	-
180	385	550	613	613	-	20	2340	5.3	29	-
180	409	525	-	1226	-	20	2340	5.3	30	-
180	375	544	869	372	-	20	2340	5.3	39	-
180	405	508	624	624	-	20	2340	5.3	31	-
180	426	494	-	1241	-	20	2340	5.3	34	-
193	350	661	1061	57	-	12	2010	10.9	40	2.9
194	350	515	1061	170	-	12	2010	10.9	38.6	2.7
196	350	368	1061	283	-	12	2010	10.9	37.6	2.6
199	158	-	1061	566	-	12	2010	10.9	38.6	2.5
158	350	693	1111	59	3.5	12	2010	10.9	53.7	3.4
163	350	536	1105	177	3.5	12	2010	10.9	51	3.3
168	350	381	1100	294	3.5	12	2010	10.9	47.8	3.1
178	350	-	1089	582	3.5	12	2010	10.9	45.1	3
137	350	713	1143	61	3.5	12	2010	10.9	64.6	4.2
139	350	555	1143	183	3.5	12	2010	10.9	65.4	4.5
143	350	395	1138	304	3.5	12	2010	10.9	63.2	3.7
150	350	-	1132	605	3.5	12	2010	10.9	63	3.4
180	281	802	-	970	-	10	2360	4.7	38.6	3.5
170	293	648	-	919	-	10	2280	6.2	38.1	3.1
165	337	841	-	879	-	10	2220	7.8	39.3	3.3
190	463	621	-	970	-	10	2360	4.7	60.1	3.8
190	500	621	-	919	3.24	10	2280	6.2	60.2	3.7
180	600	567	-	879	5.04	10	2220	7.8	62.8	3.7
220	537	693	782	138	-	20	2330	4.4	50.8	-
220	537	693	644	276	-	20	2330	4.4	44.9	-
220	537	693	506	414	-	20	2330	4.4	44.6	-
220	537	693	368	552	-	20	2330	4.4	42.4	-
220	537	693	782	138	-	20	2370	4	54	-
220	537	693	644	276	-	20	2370	4	56	-
220	537	693	506	414	-	20	2370	4	54.4	-
220	537	693	368	552	-	20	2370	4	40.6	-
220	537	693	782	138	-	20	2390	3.6	55.2	-
220	537	693	644	276	-	20	2390	3.6	53.5	-
220	537	693	506	414	-	20	2390	3.6	56.9	-
220	537	693	368	552	-	20	2390	3.6	54.7	-
220	537	693	782	138	-	20	2320	4.6	50.5	-
220	537	693	644	276	-	20	2320	4.6	48.9	-
220	537	693	506	414	-	20	2320	4.6	45.8	-
220	537	693	368	552	-	20	2320	4.6	40	-
220	537	693	782	138	-	20	2390	3.7	54.4	-
220	537	693	644	276	-	20	2390	3.7	50.2	-
220	537	693	506	414	-	20	2390	3.7	49.5	-
220	537	693	368	552	-	20	2390	3.7	40.4	-
220	537	693	782	138	-	20	2390	3.5	45	-
220	537	693	644	276	-	20	2390	3.5	46.9	-
220	537	693	506	414	-	20	2390	3.5	51.4	-
220	537	693	368	552	-	20	2390	3.5	53.2	-
220	537	693	782	138	-	20	2380	3.8	55.3	-
220	537	693	644	276	-	20	2380	3.8	55.9	-
220	537	693	506	414	-	20	2380	3.8	52.6	-
220	537	693	368	552	-	20	2380	3.8	48	-
220	537	693	782	138	-	20	2380	3.8	49.1	-
220	537	693	644	276	-	20	2380	3.8	49.9	-
220	537	693	506	414	-	20	2380	3.8	50.3	-
220	537	693	368	552	-	20	2380	3.8	47.5	-
220	537	693	782	138	-	20	2400	3.5	43.2	-
220	537	693	644	276	-	20	2400	3.5	53.7	-
220	537	693	506	414	-	20	2400	3.5	50	-
220	537	693	368	552	-	20	2400	3.5	43.3	-
220	537	693	782	138	-	20	2370	4	52.9	-
220	537	693	644	276	-	20	2370	4	49.9	-
220	537	693	506	414	-	20	2370	4	53.7	-
220	537	693	368	552	-	20	2370	4	46	-
206	413	606	-	987	-	25	2452	4.1	51	-
206	413	606	-	987	-	25	2452	4.1	49	-
206	413	606	-	987	-	25	2452	4.1	48	-
206	413	606	537	494	-	25	2452	4.1	51	-
206	413	606	537	494	-	25	2452	4.1	51	-
206	413	606	537	494	-	25	2452	4.1	51	-
206	413	606	805	245	-	25	2452	4.1	52	-
206	413	606	805	245	-	25	2452	4.1	50	-
206	413	606	805	245	-	25	2452	4.1	49	-
145.6	520	577.2	-	1040	-	25	2260	7.5	38.3	-
145.6	520	577.2	-	1040	-	25	2260	7.5	32.9	-
119.6	520	577.2	-	1040	-	25	2260	7.5	33.2	-
146.2	430	653.6	-	1032	-	25	2260	7.5	31.3	-
146.2	430	653.6	-	1032	-	25	2260	7.5	28.4	-
120.4	430	653.6	-	1032	-	25	2260	7.5	28	-
145.77	339	728.85	-	1050.9	-	25	2260	7.5	26.5	-
145.77	339	728.85	-	1050.9	-	25	2260	7.5	23.3	-
118.65	339	728.85	-	1050.9	-	25	2260	7.5	21.6	-
144.06	294	767.34	-	1029	-	25	2260	7.5	21.6	-
144.06	294	767.34	-	1029	-	25	2260	7.5	18	-
117.6	294	767.34	-	1029	-	25	2260	7.5	18.8	-
146.91	249	804.27	-	1045.8	-	25	2260	7.5	16.1	-
146.91	249	804.27	-	1045.8	-	25	2260	7.5	13.4	-
119.52	249	804.27	-	1045.8	-	25	2260	7.5	13.9	-
179	275	878	735	184	-	20	2320	5.3	41	2.8
179	275	849	455	455	-	20	2320	5.3	44	3.1
179	275	868	-	830	-	20	2320	5.3	45	2.4
190	380	744	757	189	-	20	2320	5.3	50.5	3.5
190	380	710	471	471	-	20	2320	5.3	45	2.7
190	380	715	-	874	-	20	2320	5.3	56	3.7
179	275	961	740	185	-	20	2320	5.3	33.5	2.5
179	275	978	408	408	-	20	2320	5.3	32	2.5
179	275	1010	-	640	-	20	2320	5.3	32	2.3
190	380	813	767	192	-	20	2320	5.3	44	2.8
190	380	822	426	427	-	20	2320	5.3	41	2.6
190	380	836	-	683	-	20	2320	5.3	41.5	2.3
179	325	799	839	210	-	20	2320	5.3	44	2.8
179	325	831	490	490	-	20	2320	5.3	41	2.7
179	325	825	-	923	-	20	2320	5.3	33.5	2.3
173	385	698	892	223	-	20	2320	5.3	53.5	3.1
173	385	742	515	515	-	20	2320	5.3	54	3.9
173	385	746	-	963	-	20	2320	5.3	40	2.4
159.6	380	862.4	489.3	489.3	5.7	20	2330	6.1	41.6	-
193.8	380	934.1	-	867.7	6.46	20	2330	6.1	31.4	-
197.6	380	862.4	489.3	489.3	5.7	20	2330	6.1	35.5	-
231.8	380	934.1	-	867.7	6.46	20	2330	6.1	26	-
167.2	380	862.4	489.3	489.3	5.7	20	2320	5.8	44.6	-
193.8	380	934.1	-	867.7	6.46	20	2320	5.8	36.7	-
235.6	380	934.1	-	867.7	6.46	20	2320	5.8	29.5	-
155.8	380	818.5	840.9	210.2	4.56	20	2360	3.9	46.1	-
159.6	380	862.4	489.3	489.3	5.7	20	2360	3.9	45.1	-
171	380	934.1	-	867.7	6.46	20	2360	3.9	42.9	-
190	380	818.5	840.9	210.2	4.56	20	2360	3.9	39.3	-
197.6	380	862.4	489.3	489.3	5.7	20	2360	3.9	39.5	-
205.2	380	934.1	-	867.7	6.46	20	2360	3.9	37.7	-
159.6	380	818.5	840.9	210.2	4.56	20	2350	4.5	48.1	-
163.4	380	862.4	489.3	489.3	5.7	20	2350	4.5	41	-
152	380	934.1	-	867.7	6.46	20	2350	4.5	38.7	-
193.8	380	818.5	840.9	210.2	4.56	20	2350	4.5	42.7	-
197.6	380	862.4	489.3	489.3	5.7	20	2350	4.5	35.4	-
190	380	934.1	-	867.7	6.46	20	2350	4.5	31.4	-
159.6	380	818.5	840.9	210.2	4.56	20	2350	4.7	48.5	-
159.6	380	862.4	489.3	489.3	5.7	20	2350	4.7	45.4	-
163.4	380	934.1	-	867.7	6.46	20	2350	4.7	37	-
197.6	380	818.5	840.9	210.2	4.56	20	2350	4.7	41.3	0-
197.6	380	862.4	489.3	489.3	5.7	20	2350	4.7	36.8	-
212.8	380	934.1	-	867.7	6.46	20	2350	4.7	31.2	-
159.8	340	556	1020	238	-	20	2336	3.6	50	-
159.8	340	556	638	596	-	20	2315	3.6	45.3	-
159.8	340	556	319	894	-	20	2295	3.6	44	-
137.1	380	927	869.2	202	-	10	2470	3.7	108	5.7
146.5	380	927	543.2	505.1	-	10	2470	3.7	104.8	5
162.3	380	927	-	1010.2	-	10	2470	3.7	108.5	5.1
138.2	380	927	869.2	195	-	10	2390	4.9	102.5	6.3
149.8	380	927	543.2	487.5	-	10	2390	4.9	103.1	5.1
170.4	380	927	-	975.1	-	10	2390	4.9	100.8	5.9
139.7	380	927	869.2	187.8	-	10	2300	5.9	104.3	5.3
153.1	380	927	543.4	469.4	-	10	2300	5.9	96.8	6.2
175	380	927	-	938.8	-	10	2300	5.9	91.2	4.2
185.4	309	864	848	211	1.0197	16	2380	6.9	42.9	-
191.7	320	817.5	538	538	1.056	16	2380	6.9	42.5	-
201.6	336	785	-	1060	1.1088	16	2380	6.9	40.9	-
192.5	386	829	808	202	2.0458	16	2380	6.9	51.6	-
200	399	795	504	504	2.1147	16	2380	6.9	51.6	-
210	420	738	-	1014	2.226	16	2380	6.9	50.3	-
205	300	697	-	1075	-	20	2450	3.1	35	2.5
205	300	697	-	1027	-	20	2370	7.1	29.2	2.4
205	300	697	-	1027	-	20	2360	7.8	27.7	1.9
180	350	706	-	1089	-	20	2450	3.1	47.6	3.4
180	350	706	-	1041	-	20	2370	7.1	42	2.6
180	350	706	-	1041	-	20	2360	7.8	42.9	2.6
185	425	696	-	1028	-	20	2450	3.1	60	3.9
185	425	696	-	982	-	20	2370	7.1	53.7	3.7
185	425	696	-	982	-	20	2360	7.8	53.2	3.4
165	485	685	-	1039	-	20	2450	3.1	78.2	4.7
165	485	685	-	979	-	20	2370	7.1	71.2	4.1
165	485	685	-	982	-	20	2360	7.8	65.4	4.2
178.3	358	730.4	783.6	299.3	0.3	19	2570	2.7	33.6	3.9
178.3	358	730.4	458.3	598.4	0.3	19	2570	2.7	30.4	3.9
178.3	358	730.4	-	1020	0.3	19	2570	2.7	29.1	3.3
195	300	787.1	756.4	189.1	-	20	2300	5.2	39.5	-
195	300	737.4	485.5	485.5	-	20	2300	5.2	40.8	-
195	300	712.6	-	951.4	-	20	2300	5.2	43.7	-
195	300	814.4	733	183.2	-	20	2300	5.5	41	-
195	300	804.2	450.7	450.7	-	20	2300	5.5	38.8	-
195	300	807.9	-	855.2	-	20	2300	5.5	39.9	-
214.2	210	929	-	966	-	22	2451	7.8	19.7	2
196	280	866	-	940	-	22	2387	6.9	35.7	2.9
161	350	858	-	974	3.5	22	2362	4.2	66.8	4.6
212.1	210	932	-	970	-	22	2456	7.5	21.8	2
193.2	280	870	-	970	-	22	2455	6.4	36.1	2.9
157.5	350	858	-	1029	3.5	22	2496	4.2	68.5	4.8
207.9	210	938	-	953	-	22	2401	7.6	21	2.1
187.6	280	877	-	988	-	22	2484	5.4	41.1	3
150.5	350	868	-	982	3.5	22	2363	3.6	70.2	4.9
205.8	210	943	-	977	-	22	2447	6.9	23.6	2.2
190.4	280	873	-	962	-	22	2458	5.8	39.7	3
157.5	350	858	-	1016	3.5	22	2464	3.9	66.5	5
179	275	878	735	184	-	19	2320	5.3	49.3	4.1
179	275	849	455	455	-	19	2320	5.3	47.5	4.7
179	275	868	-	830	-	19	2320	5.3	53.7	4.9
190	380	714	757	189	-	19	2320	5.3	64.8	4.7
190	380	710	471	471	-	19	2320	5.3	63.5	4.8
190	380	715	-	874	-	19	2320	5.3	65.1	5
179	275	961	740	185	-	19	2320	5.3	64.8	2.5
179	275	978	408	408	-	19	2320	5.3	63.5	2.4
179	275	1010	-	640	-	19	2320	5.3	65.1	2.3
190	380	813	767	192	-	19	2320	5.3	54.9	3.2
190	380	822	426	427	-	19	2320	5.3	51.5	2.7
190	380	836	-	683	-	19	2320	5.3	50.3	2.4
179	325	799	839	210	-	19	2320	5.3	56.5	2.9
179	325	831	490	490	-	19	2320	5.3	48.9	2.6
179	325	825	-	923	-	19	2320	5.3	43.1	2.4
173	385	698	892	233	-	19	2320	5.3	67.4	3.5
173	385	742	515	515	-	19	2320	5.3	61.2	2.9
173	385	746	-	963	-	19	2320	5.3	53.7	2.5
